# Expression of Concern for Huang et al., “Long-Read Metagenomics of Marine Microbes Reveals Diversely Expressed Secondary Metabolites”

**DOI:** 10.1128/spectrum.01851-24

**Published:** 2024-08-22

**Authors:** American Society for Microbiology

## EXPRESSION OF CONCERN

The American Society for Microbiology (ASM) and *Microbiology Spectrum* are issuing this Expression of Concern regarding the following publication:

Huang R, Wang Y, Liu D, Wang S, Lv H, Yan Z. 2023. Long-read metagenomics of marine microbes reveals diversely expressed secondary metabolites. Microbiol Spectr 11:e01501-23. https://doi.org/10.1128/spectrum.01501-23.

*Microbiology Spectrum* was notified by a concerned reader that the article shares significant structure and text with an article published in ISME J:

Waschulin V, Borsetto C, James R, Newsham KK, Donadio S, Corre C, Wellington E. 2022. Biosynthetic potential of uncultured Antarctic soil bacteria revealed through long-read metagenomic sequencing. ISME J 16:101-111. https://doi.org/10.1038/s41396-021-01052-3.

ASM is independently verifying these concerns and has initiated an inquiry with the authors. In publishing this Expression of Concern, ASM is following its Publishing Ethics Policies and Procedures and, as a member of the Committee on Publication Ethics (COPE), follows its guidelines in this regard. ASM and *Microbiology Spectrum* have requested the corresponding authors to provide us with an explanation soon. This Expression of Concern could be updated accordingly thereafter.

